# The *N*-Acetylmuramic Acid 6-Phosphate Phosphatase MupP Completes the *Pseudomonas* Peptidoglycan Recycling Pathway Leading to Intrinsic Fosfomycin Resistance

**DOI:** 10.1128/mBio.00092-17

**Published:** 2017-03-28

**Authors:** Marina Borisova, Jonathan Gisin, Christoph Mayer

**Affiliations:** Microbiology/Biotechnology, Department of Biology, Interfaculty Institute of Microbiology and Infection Medicine, University of Tübingen, Tübingen, Germany; University of Chicago

## Abstract

Bacterial cells are encased in and stabilized by a netlike peptidoglycan (PGN) cell wall that undergoes turnover during bacterial growth. PGN turnover fragments are frequently salvaged by the cells via a pathway referred to as PGN recycling. Two different routes for the recycling of the cell wall sugar *N*-acetylmuramic acid (MurNAc) have been recognized in bacteria. In *Escherichia coli* and related enterobacteria, as well as in most Gram-positive bacteria, MurNAc is recovered via a catabolic route requiring a MurNAc 6-phosphate etherase (MurQ in *E. coli*) enzyme. However, many Gram-negative bacteria, including *Pseudomonas* species, lack a MurQ ortholog and use an alternative, anabolic recycling route that bypasses the *de novo* biosynthesis of uridyldiphosphate (UDP)-MurNAc, the first committed precursor of PGN. Bacteria featuring the latter pathway become intrinsically resistant to the antibiotic fosfomycin, which targets the *de novo* biosynthesis of UDP-MurNAc. We report here the identification and characterization of a phosphatase enzyme, named MupP, that had been predicted to complete the anabolic recycling pathway of *Pseudomonas* species but has remained unknown so far. It belongs to the large haloacid dehalogenase family of phosphatases and specifically converts MurNAc 6-phosphate to MurNAc. A Δ*mupP* mutant of *Pseudomonas putida* was highly susceptible to fosfomycin, accumulated large amounts of MurNAc 6-phosphate, and showed lower levels of UDP-MurNAc than wild-type cells, altogether consistent with a role for MupP in the anabolic PGN recycling route and as a determinant of intrinsic resistance to fosfomycin.

## IMPORTANCE

Many Gram-negative bacteria, but not *E. coli*, make use of a cell wall salvage pathway that contributes to the pool of UDP-MurNAc, the first committed precursor of cell wall synthesis in bacteria. This salvage pathway is of particular interest because it confers intrinsic resistance to the antibiotic fosfomycin, which blocks *de novo* UDP-MurNAc biosynthesis. Here we identified and characterized a previously missing enzyme within the salvage pathway, the MurNAc 6-phosphate phosphatase MupP of *P. putida*. MupP, together with the other enzymes of the anabolic recycling pathway, AnmK, AmgK, and MurU, yields UDP-MurNAc, renders bacteria intrinsically resistant to fosfomycin, and thus may serve as a novel drug target for antimicrobial therapy.

## INTRODUCTION

Bacterial cells are surrounded by a rigid peptidoglycan (PGN) structure that protects the cell membrane from rupture because of the high intracellular turgor pressure and stabilizes the bacterial cell against adverse effects of the environment (1, 2). The PGN is a wide-meshed netlike polymer composed of linear glycan strands, consisting of the amino sugars *N*-acetylglucosamine (GlcNAc) and *N*-acetylmuramic acid (MurNAc), which are cross-linked by peptides. Despite the cell-stabilizing and shape-maintaining functions of PGN, it is a highly dynamic structure that permanently undergoes remodeling and turnover during bacterial growth. PGN fragments released thereby are frequently salvaged by the cells via a pathway referred to as PGN recycling. PGN recycling has attracted much attention because of its connection to β-lactam antibiotic resistance (3–5) and the host innate immune response, as well as bacterial differentiation and survival (6–8).

**FIG 1:**
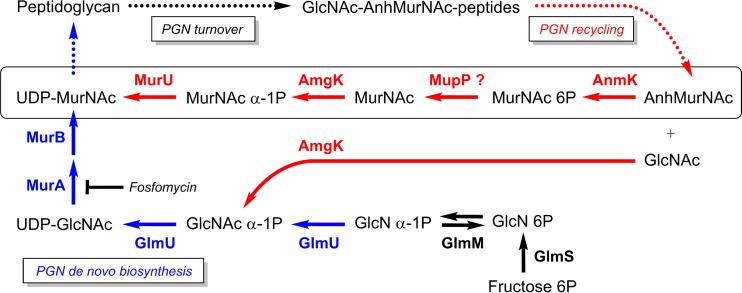
Simplified scheme of the PGN *de novo* biosynthesis, turnover, and recycling metabolic pathways of *P. putida*. The main PGN turnover products GlcNAc-anhMurNAc-peptides (also called anhydro-muropeptides) are formed by periplasmic autolytic enzymes and are reutilized in the process of PGN recycling, which is in red. The transport of anhydro-muropeptides by the permease AmpG and their processing within the cytoplasm, yielding anhMurNAc, GlcNAc, and peptides by the *N*-acetylglucosaminidase NagZ, the anhMurNAc-peptide amidase (AmpD), and the l,d-carboxypeptidase LdcA, are conserved within Gram-negative bacteria. Also, the anhMurNAc kinase (AnmK) that phosphorylates anhMurNAc, yielding MurNAc 6P, is conserved. However, in *P. putida*, MurNAc 6P is then recycled, specifically involving an alternative, anabolic recycling route (22), converting MurNAc 6P to UDP-MurNAc. One step of this pathway, the dephosphorylation of MurNAc 6P by a still unknown MurNAc 6P phosphatase that we named MupP, has remained unknown so far. Its product, however, MurNAc, is known to be phosphorylated by the anomeric MurNAc and GlcNAc kinase AmgK, yielding MurNAc α-1P and finally the MurU enzyme that catalyzes the uridylation of MurNAc α-1P to UDP-MurNAc. Thus, the anabolic recycling route provides a shortcut that bypasses the *de novo* PGN biosynthesis pathway, which is in blue. This recycling pathway, including the enzymes MupP, AmgK, and MurU, leads to increased intrinsic resistance to the antibiotic fosfomycin.

In Gram-negative bacteria, the PGN cell wall is steadily dismantled (PGN turnover) by the action of lytic transglycosylases and endopeptidases (9). These potentially autolytic enzymes (autolysins) release anhydro-muropeptides (GlcNAc–1,6-anhydro-MurNAc [GlcNAc-anhMurNAc-peptides]) from the cell wall (Fig. 1) that contain 1,6-anhydro-MurNAc (anhMurNAc), a unique, nonreducing form of the cell wall sugar MurNAc. Anhydro-muropeptides are instantly recovered (PGN recycling) by a set of conserved recycling proteins best studied in the Gram-negative model organism *Escherichia coli*, which reportedly recycles about 45% of its PGN wall in each generation (9, 10). The transporter AmpG, initially identified as a positive effector of AmpC β-lactamase induction, takes up GlcNAc-anhMurNAc(-peptides) (9, 11, 12), which is further hydrolyzed by a negative regulator of AmpC induction in the cytoplasm, the anhydromuramyl-l-alanine amidase AmpD (3, 13), as well as by the *N*-acetylglucosaminidase NagZ (11, 14) and l,d-carboxypeptidase LdcA (15) recycling enzymes, together yielding GlcNAc, anhMurNAc, l-alanine-*iso*-d-glutamate-*meso*-diaminopimelate tripeptide, and d-alanine (d-Ala) (9, 16). The tripeptide is directed to PGN biosynthesis through ligation onto UDP-MurNAc, and d-Ala is self-ligated to yield d-Ala–d-Ala and further to form UDP-MurNAc-pentapeptide (9, 17). In *E. coli*, GlcNAc is phosphorylated by the kinase NagK (18), yielding GlcNAc-6-phosphate (GlcNAc 6P), and anhMurNAc is phosphorylated by AnmK, yielding MurNAc 6-phosphate (MurNAc 6P) (19, 20), which subsequently is catabolically cleaved, forming GlcNAc 6P and d-lactic acid, by the MurNAc 6P etherase MurQ (catabolic recycling route) (21).

In *Pseudomonas* species, AnmK is generally present; however, the NagK and MurQ enzymes are missing. We recently revealed an alternative PGN recycling pathway (anabolic recycling route) in *Pseudomonas putida* that is broadly conserved in Gram-negative bacteria but absent in *E. coli* and related enterobacteria (22, 23). This anabolic recycling route involves a sugar kinase, AmgK, that generates MurNAc α-1-phosphate (MurNAc α-1P) and a uridylyltransferase, MurU, that subsequently converts the latter to UDP-MurNAc (22) (Fig. 1). This anabolic recycling route bypasses the classical *de novo* biosynthesis of UDP-MurNAc, which relies on UDP-GlcNAc by enol-pyruvyltransferase MurA and reducing flavoenzyme MurB in almost all bacteria, together yielding UDP-MurNAc (Fig. 1). Fosfomycin, an antibiotic that inhibits the *de novo* biosynthesis of UDP-MurNAc by covalently binding to the MurA enzyme (24), is less effective in bacteria that contain the anabolic PGN recycling pathway (22). Up to 8-fold-increased fosfomycin susceptibility was determined in PGN recycling mutants of *Pseudomonas aeruginosa* (23). Still, the anabolic PGN recycling in *Pseudomonas* contains one unknown enzyme, most likely a putative MurNAc 6P phosphatase (MupP) that catalyzes the conversion of MurNAc 6P to MurNAc (Fig. 1) (22). In this report, we describe the identification and biochemical characterization of this missing recycling enzyme, MupP (PP_1764). Investigation of the accumulation of metabolites in a Δ*mupP* mutant by mass spectrometry (MS) confirmed the role of MupP in PGN recycling and provides a rationale for its role in fosfomycin resistance. Notably, the enzyme MupP (PA3172) was independently discovered in *P. aeruginosa* by genetic screening by the group of Thomas Bernhardt. They additionally link MupP and PGN recycling with a modulating influence on AmpC β-lactamase resistance (25).

## RESULTS

### Detection of MurNAc 6P phosphatase activity in *P. putida* cell extracts.

**FIG 2:**
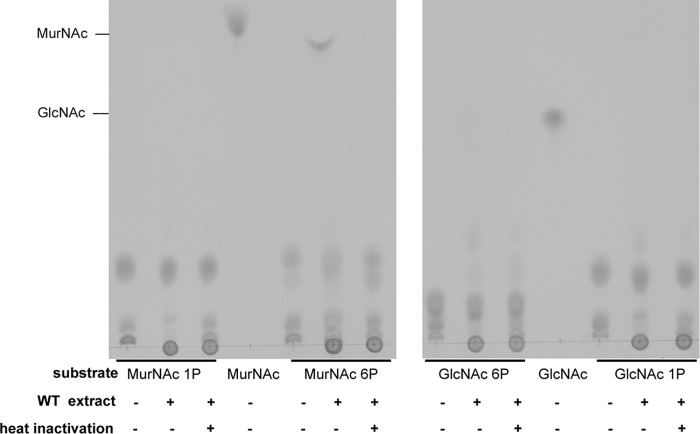
Identification of MurNAc 6P phosphatase activity in *P. putida* cell extract. The sugars MurNAc 1P and MurNAc 6P (left) and the sugars GlcNAc 6P and GlcNAc 1P (right) were generated enzymatically from MurNAc and GlcNAc substrates, respectively, with MurK kinase for C6 phosphorylation and with AmgK kinase for C1 phosphorylation. Ten-microliter volumes of the phosphorylated sugars were mixed with 10 µl of cell extract from *P. putida* KT2440 cells (wild-type [WT] extract). As an additional control, the WT extract was heat inactivated. After 24 h of incubation at 37°C, two 2.5-µl samples, as well as MurNAc and GlcNAc standards, both at 50 mM, were spotted onto the TLC plate, separated on the mobile phase, dehydrated in a sulfuric acid bath, and visualized by charring at 180°C.

We showed previously that MurNAc accumulates in a Δ*amgK* mutant of *P. putida*, while MurNAc 6P is the product of an AnmK reaction (22). Therefore, we concluded that a hypothetical MurNAc 6P phosphatase, which we named MupP, is required to convert MurNAc 6P to MurNAc to complete the anabolic PGN recycling pathway (Fig. 1). To test whether this enzyme exists, we incubated extracts of wild-type *P. putida* cells with MurNAc 6P and analyzed the phosphatase reaction by thin-layer chromatography (TLC) (Fig. 2). In addition, we tested MurNAc α-1P, GlcNAc 6P, and GlcNAc α-1-phosphate (GlcNAc α-1P) as possible substrates. We harvested *P. putida* cells at an optical density (OD) of 1, since the PGN recycling enzymes in Gram-negative bacteria, including MupP, were expected to be active in late exponential growth phase (9, 10). We further used a large amount (25 µg) of cell extract and 24 h of incubation at 37°C to ensure substrate conversion even with the expected low concentration of the MupP enzyme in the bacterial extract. Indeed, a spot appeared on the TLC plate with a retention time for MurNAc when MurNAc 6P was incubated with the cell extract. This MurNAc spot was absent when the cell extract was heat treated, which implies that MurNAc 6P is dephosphorylated by a heat-sensitive enzyme present in the extract. This enzyme apparently displays narrow substrate specificity, as MurNAc was not detected when the substrate MurNAc α-1P was used and GlcNAc did not appear with GlcNAc 6P or GlcNAc α-1P. When high-contrast and low-brightness adjustments were used, however, a spot with a retention similar to that of GlcNAc was slightly visible when the substrate GlcNAc 6P was used (data not shown). These results indicated the presence of a MupP phosphatase in *P. putida*.

### Identification of MupP candidates by bioinformatic tools.

We had previously identified the nucleotidyltransferase MurU of the anabolic recycling pathway of *P. putida* by using bioinformatic analysis based on amino acid sequence identity with the transferase domain of the UDP-GlcNAc-forming enzyme GlmU (22). Searching pseudomonad genomes for a so far uncharacterized and highly conserved putative GlmU-like nucleotidyltransferase that is not present in the genomes of *E. coli* and related members of the family *Enterobacteriaceae* led directly to the discovery of *murU* (*pp0406*) and the adjacent gene *pp0405* (*amgK*) (22). The MurNAc-6P phosphatase of *P. putida*, however, could not be identified by a similar approach because phosphatases belong to very different protein families and *mupP* could be a member of any of these. However, we have recognized in our studies that in proteobacteria, particularly *Betaproteobacteria* and *Gammaproteobacteria*, either the catabolic MurQ or the anabolic AmgK-MurU recycling pathway is present (cf. reference 22 and the supplemental material for that reference). Furthermore, a specific MurNAc 6P phosphatase is likely not present in *E. coli*, since a *murQ* mutant of MG1655 accumulates large amounts of MurNAc 6P (8, 21). Thus, we proposed that if *mupP* frequently co-occurs with *amgK* and is absent when *murQ* is present, we could identify this enigmatic phosphatase by a multigenome comparison. Therefore, we used a bioinformatic tool, the phylogenetic profiler for single genes provided by the Integrated Microbial Genomes & Microbiomes database. Representative results obtained with this tool in genome comparisons for the identification of MupP candidates are summarized in Table 1. Several analyses were conducted with the *P. putida* KT2440 genome as the reference and various *Pseudomonadales*, *Gammaproteobacteria*, and *Betaproteobacteria* genomes as probes. Choosing five genomes and using a cutoff of 30% amino acid sequence identity gave optimal results. This cutoff was high enough to exclude most of the paralogs but kept the majority of homologous proteins in the classes *Betaproteobacteria* and *Gammaproteobacteria*. We then excluded all of the proteins that were found in *E. coli*. This resulted in a list of 33 to 109 candidate proteins, and the lists always contained AmgK and MurU, as expected. Three putative phosphatases, with the locus tags PP_1764, PP_1907, and PP_5147, showed up several times in different comparisons. They are annotated as haloacid dehalogenase (HAD) phosphatases of the HAD_2 protein family (Pfam) group. The HAD superfamily is a huge enzyme family containing phosphosugar phosphatases but shows only low sequence conservation among its members and, besides phosphatases, also contains enzymes with ATPase, phosphomutase, phosphonohydrolase, and HAD activities. Not all of these HAD proteins showed up in every run. When only genomes from the group of *Betaproteobacteria* were compared in the analysis, PP_1907 did not show up and when only genomes from the group of *Gammaproteobacteria* were used, PP_5147 was not found (Table 1). We proposed that a HAD family phosphatase would be a good MupP candidate.

**TABLE 1:** Representative results of bioinformatic analysis for identification of MupP candidates

Multigenome comparison^a^ with *P. putida* KT2440 reference genome	No. of protein matches^b^	HAD-like phosphatase candidate(s)
*Pseudomonadales^c^*	94	PP_1764, PP_1907, PP_5147
*Gammaproteobacteria^d^*	65	PP_1764, PP_1907
*Gammaproteobacteria^e^*	48	PP_1764, PP_1907
*Gammaproteobacteria^f^*	33	PP_1764
*Betaproteobacteria^g^*	109	PP_1764, PP_5147

^a^ All selected genomes contain *amgK* and *murU* orthologs.

^b^ *E. coli* matches were excluded; *amgK* and *murU* were found.

^c^ *Acinetobacter baumannii* 1656-2, *Azotobacter vinelandii* CA, *Cellvibrio japonicus* Ueda107, *Pseudomonas fluorescens* Pf0-1, and *Psychrobacter arcticus* 273-4.

^d^ *Aeromonas salmonicida. salmonicida* A449, *Hahella chejuensis* KCTC 2396, *Idiomarina loihiensis* L2TR, *Shewanella denitrificans* OS217, and *Thioalkalimicrobium aerophilum* AL3.

^e^ *Kangiella geojedonensis* KCTC 23420, *Saccharophagus degradans* 2-40, *Thioalkalivibrio paradoxus* ARh 1, *Xanthomonas oryzae* pv. *oryzae* PXO99A, and *Acidithiobacillus ferrivorans* SS3.

^f^ *Halomonas elongata* DSM 2581, *Xylella fastidiosa* subsp. *fastidiosa* GB514, *Thiomicrospira crunogena* XCL-2, *Shewanella baltica* OS117, and *Marinobacter aquaeolei* VT8.

^g^ *Azoarcus* sp. strain BH72, *Burkholderia mallei* ATCC 23344, *Nitrosomonas europaea* ATCC 1971, *Ralstonia eutropha* H16, and *Thiobacillus denitrificans* ATCC 25259.

### Fosfomycin hypersensitivity of *P. putida* Δ*pp_1764* (Δ*mupP*) mutant.

**FIG 3:**
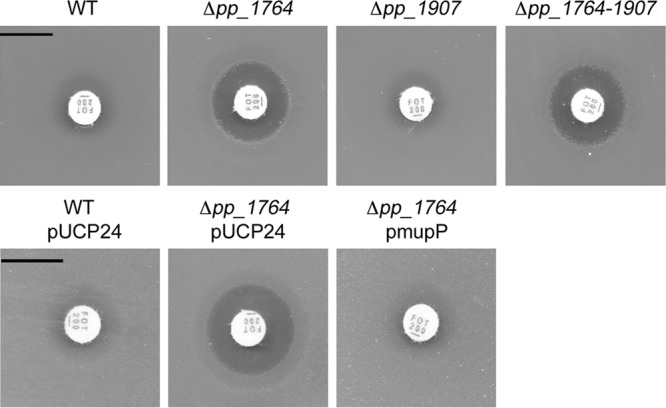
Fosfomycin susceptibility testing of two putative MurNAc 6P phosphatase mutants (top). LB solid agar plates were overlaid with soft agar mixed with 10-µl overnight cultures of wild-type (WT) *P. putida* and the Δ*pp_1764*, Δ*pp_1907*, and Δ*pp_1764*_*1907* phosphatase deletion mutants (bottom). *P. putida* WT and Δ*pp_1764* mutant strains were transformed with empty plasmid pUCP24 and complementation plasmid p*mupP*. To maintain the plasmids, LB plates and soft agar were supplemented with gentamicin. Finally, 200-µg fosfomycin discs were added to each agar plate and the inhibition zones were determined after 16 h of incubation at 30°C. Scale bars, 1 cm.

We previously showed that deletion of the recycling genes *amgK* and *murU* leads to increased susceptibility to the antibiotic fosfomycin in *P. putida* and *P. aeruginosa* (22, 23). We assumed that the inactivation of MupP function would have the same phenotype. Therefore, we constructed *P. putida* deletion mutants of candidate genes *pp_1764* and *pp_1907* as described in Tables S1 and S2 in the supplemental material and tested both mutants for susceptibility to fosfomycin with agar diffusion assays (Fig. 3). Indeed, the Δ*pp_1764* mutant turned out to be fosfomycin hypersensitive, whereas the wild-type and Δ*pp_1907* mutant *P. putida* strains were resistant to the drug. Also, the Δ*pp_1764* Δ*pp_1907* double mutant showed fosfomycin sensitivity with an inhibition zone similar in size to that of the Δ*pp_1764* mutant. Expression of PP1764 from the *E. coli*-*Pseudomonas* pUCP24 shuttle vector under the control of a *lac* promoter (see Tables S1 and S2), completely restored fosfomycin resistance (Fig. 3). Antibiotic susceptibility was not affected when the wild-type and Δ*mupP* mutant strains were transformed with the empty pUCP24 plasmid. Thus, we could show by fosfomycin susceptibility testing that *pp_1764* most likely encodes the missing recycling enzyme MupP; we use this designation further on.

10.1128/mBio.00092-17.6 TABLE S1  Oligonucleotides used in this study. Download TABLE S1, DOCX file, 0.03 MB. Copyright © 2017 Borisova et al. 2017 Borisova et al. This content is distributed under the terms of the Creative Commons Attribution 4.0 International license.

10.1128/mBio.00092-17.7 TABLE S2  Strains and plasmids used in this study. Download TABLE S2, DOCX file, 0.04 MB. Copyright © 2017 Borisova et al. 2017 Borisova et al. This content is distributed under the terms of the Creative Commons Attribution 4.0 International license.

### Accumulation of MurNAc 6P and decreased levels of UDP-GlcNAc and UDP-MurNAc in Δ*mupP* mutant.

**FIG 4:**
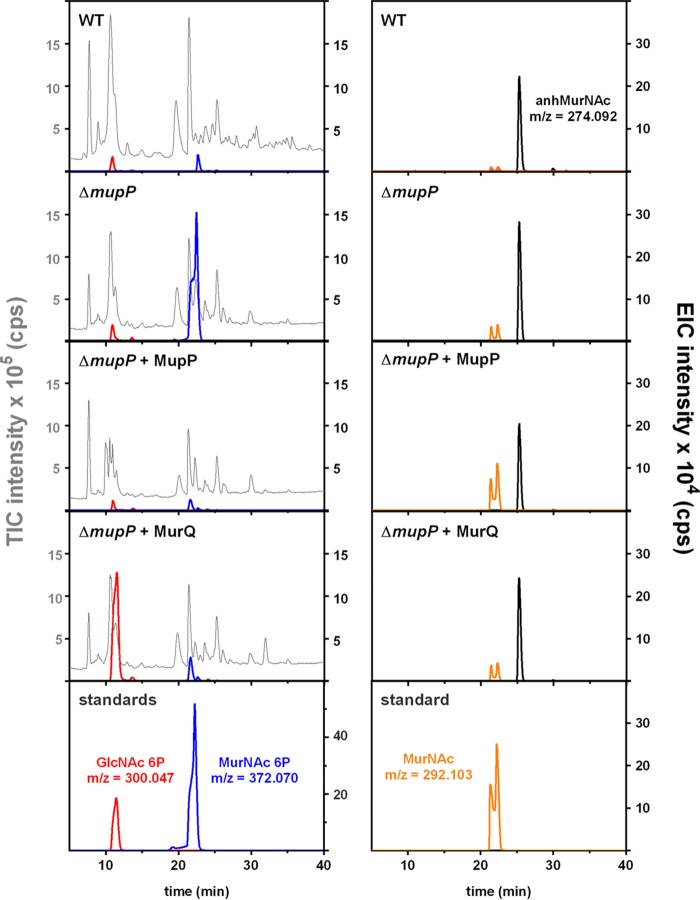
LC-MS analysis of the accumulation of recycling products in the wild type (WT) and recycling mutant strains. Cytosolic fractions of wild-type and Δ*mupP* mutant *P. putida* strains were prepared as previously described (22). In addition, 100 µl of cytosolic fractions of the Δ*mupP* was incubated with 2.5 µg of the *P. putida* MupP (Δ*mupP* + MupP) or *N*-acetylmuramic acid 6P etherase MurQ (Δ*mupP* + MurQ) recombinant enzyme for 3 h at 37°C prior to measurements. A 3-µl portion of each sample was injected into the Gemini C_18_ column (150 by 4.6 mm, 110 Å, 5 μm; Phenomenex), and LC-MS analysis was performed as previously described (22). Shown are the TIC intensity (10^5^ counts/s [cps], in gray) and EIC intensity (10^4^ cps) of GlcNAc 6P (theoretical *m/z* = 300.048; measured *m/z* = 300.047; in red), MurNAc 6P (*m/z* = 372.070; in blue), MurNAc (*m/z* = 292.103; in orange) and for anhMurNAc (theoretical *m/z* = 274.093; measured *m/z* = 274.092; in black) of bacterial samples and standards.

We investigated by liquid chromatography (LC)-MS whether recycling intermediates accumulate specifically in the cytosolic fractions of Δ*mupP* (Δ*pp_1764*) mutant cells and not in wild-type cells (Fig. 4). The Δ*mupP* recycling mutant accumulated a metabolite with a retention time of 22 min and an exact mass in negative-ion mode (*m/z* = 372.070) that is identical to the exact mass of MurNAc P. Only very small amounts of MurNAc P were found in the cytosol of wild-type cells. Furthermore, when the cytosolic extract of Δ*mupP* cells was incubated with MupP recombinant enzyme, the signal of MurNAc P disappeared and a signal with *m/z* = 292.103 appeared, which is in agreement with the expected theoretical mass of MurNAc (M-H)^−^. The appearance of two signals is due to the α- and β-anomeric isoforms of MurNAc under the LC conditions applied, as also observed for the MurNAc standard. This indicates that MupP indeed possesses phosphatase activity and possibly converts MurNAc 6P to MurNAc (Fig. 4). However, different isoforms of phosphosugars cannot be distinguished by their exact masses. Thus, we treated the Δ*mupP* extract with MurQ etherase, which specifically converts MurNAc 6P to GlcNAc 6P. In the presence of MurQ, the signal with *m/z* = 372.070 disappeared and a new signal with *m/z* = 300.047 (of GlcNAc 6P) appeared. This indicated that Δ*mupP* cells indeed accumulate MurNAc 6P, as the compound can be cleaved by the MurNAc 6P-specific etherase MurQ and GlcNAc 6P (expected exact mass in the negative-ion mode, *m/z* = 300.048) is formed (Fig. 4). In addition, we showed that similar amounts of metabolites were extracted from all of the samples, as seen by comparable intensities of the total ion chromatograms (TICs) and by similar amounts of intracellular anhMurNAc (exact *m/z* = 274.092) (Fig. 4).

**FIG 5:**
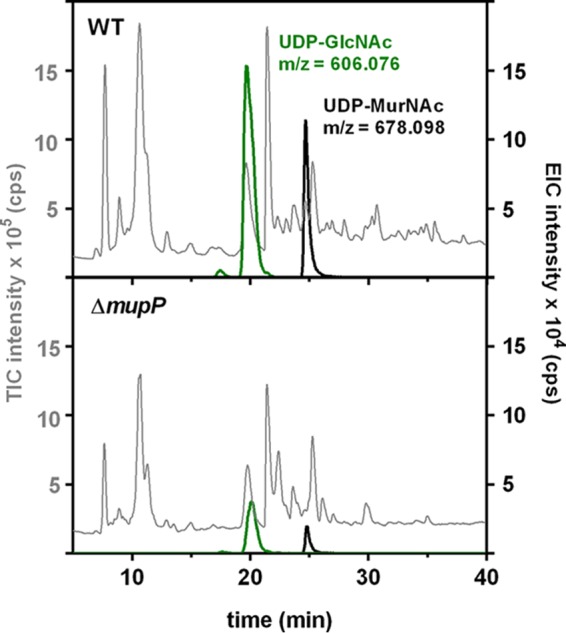
LC-MS analysis of the accumulation of PGN precursors in the wild type (WT) and recycling mutants. Cytosolic fractions of *P. putida* wild-type and Δ*mupP* mutant strains were analyzed by LC-MS in negative-ion mode by using parameters as described above. Chromatograms of the samples investigated are presented as the TIC intensity (10^5^ counts/s [cps]; in gray) and EIC intensity (10^4^ cps) of UDP-GlcNAc (theoretical *m/z* = 606.073; measured *m/z* = 606.076; in green) and UDP-MurNAc (theoretical *m/z* = 678.095; measured *m/z* = 678.098; in black).

The PGN recycling pathway, including the AmgK, MurU, and MupP enzymes, is linked with the *de novo* PGN biosynthesis pathway leading to UDP-MurNAc and causes hypersensitivity to fosfomycin. Therefore, we aimed to show the direct influence that blocking of recycling has on the pool of PGN precursors (Fig. 5). Interestingly, in extracts of the Δ*mupP* mutant, we found UDP-GlcNAc and UDP-MurNAc in amounts that were four and six times, respectively, lower than those in the wild type.

### Determination of kinetic parameters and substrate specificity of recombinant MupP.

Recombinant *P. putida* MupP was heterologously expressed in *E. coli* as a C-terminally His_6_-tagged fusion protein with plasmid pJGK84, as indicated in Table S1. A large amount of pure protein (19.2 mg) was obtained from 2 liters of culture and purified by Ni^2+^ affinity and gel filtration chromatography. Protein purity and size (calculated, 25.89 kDa) were monitored by SDS-PAGE (see Fig. S1). The long-term stability of the MupP enzyme in solution at −80°C was increased by the addition of 20% glycerol.

10.1128/mBio.00092-17.2 FIG S1  Analysis of MupP overexpression and purity by SDS-PAGE and Coomassie brilliant blue staining. The protein was overproduced in *E. coli* BL21(DE3) cells carrying plasmid pET29-*mupP* (p*mupP*). Lanes: 1, 20- to 120-kDa protein molecular size standards; 2, *E. coli* cell extract before IPTG induction; 3, *E. coli* cell extract after 0.1 mM IPTG induction for MupP overexpression; 4, 25 μg of purified MupP enzyme. The exact size of MupP is 25.89 kDa. Download FIG S1, TIF file, 1.1 MB. Copyright © 2017 Borisova et al. 2017 Borisova et al. This content is distributed under the terms of the Creative Commons Attribution 4.0 International license.

**FIG 6:**
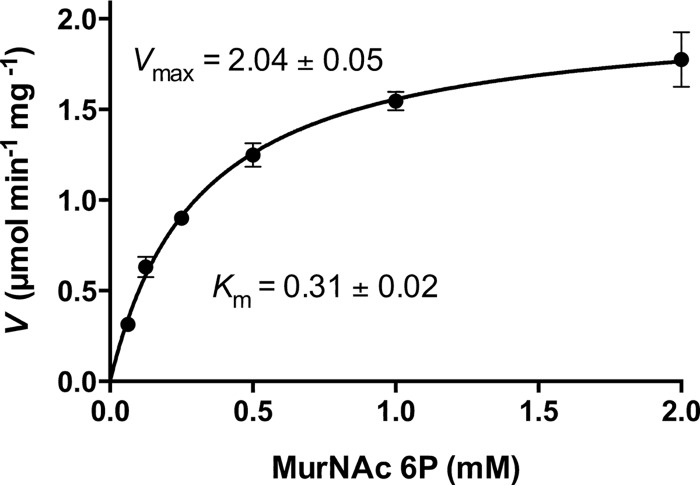
MupP kinetic parameters. Kinetic parameters were measured in 33 mM phosphate buffer (pH 7.6) with 10 mM MgCl_2_ at 22°C. To determine the *K*_*m*_ (mM) and *V*_max_ (µmol min^−1^ mg^−1^), different amounts of MurNAc 6P (0.0625 to 2 mM) were incubated with 19.31 nM (0.5 mg/liter) recombinant MupP enzyme for 2.5 min. Reactions were stopped, and MurNAc release was detected by MS. Data are presented as means of three independent experiments ± the standard deviations.

The high-performance liquid chromatography (HPLC) column and the buffer conditions used before for analysis of intracellular metabolites in cytosolic fractions were not optimal for the determination of kinetic parameters because of the poor efficiency of MurNAc and MurNAc 6P separation (cf. Fig. 4). Therefore, we developed a new method that allowed the separation of these two phosphosugars with retention times of 5 and 17 min, respectively (see Fig. S2). This analysis allowed the quantification of MurNAc, the product of the MupP reaction, and the determination of the kinetic parameters of the phosphatase with the substrate MurNAc 6P (S. Unsleber, M. Borisova, and C. Mayer, unpublished data). We observed very low MupP activity in the absence of Mg^2+^ ions, but when the reaction buffer was supplemented with 10 mM MgCl_2_, MurNAc production was increased 16.4 times (see Fig. S2). We also determined the optimal temperature and pH conditions for kinetic experiments. Recombinant MupP enzyme lost about 10% of its activity at 22°C and 70% at 37°C within 40 min (see Fig. S3). The optimum temperature was 45°C; however, at that temperature, MupP was completely inactivated within 40 min. About 50% of its activity remained at 22°C, and about 85% of its activity remained at 37°C. Since the enzyme was more stable at 22°C than at 37°C, we chose 22°C as the assay temperature and reduced the reaction time for kinetic experiments to 2.5 min. Moreover, protein stability and activity were optimal at pH 6 to 9. At pH 4 and pH 10, its activity was only 40 and 60%, respectively, of the maximum (see Fig. S3). Therefore, a phosphate buffer with a pH of 7.6 containing 10 mM MgCl_2_ was used to perform MupP kinetic experiments. To quantify the amount of MurNAc released by MupP with MurNAc 6P as the substrate in a concentration range of 0.00625 to 2 mM, we additionally included a standard curve for MurNAc (0.6 to 78 pmol). At higher concentrations of MurNAc, a nonlinear dependency of the concentration and the area under the curve (AUC) in extracted ion chromatograms (EICs) for MurNAc was observed (see Fig. S4). Therefore, we reduced the product yield in the enzyme assays by lowering the enzyme concentration (19.31 nM) and reaction time (2.5 min) at 22°C, which allowed us to quantify the MurNAc product within the linear range of the standard curve. MupP activity was calculated by determining the AUC of the EIC for MurNAc (*m/z* = 292.102) of MupP reactions and standards. A *K*_*m*_ value of 310 ± 20 μM and a *V*_max_ value of 2.04 ± 0.05 μmol min^−1^ mg^−1^ were obtained for MurNAc 6P (Fig. 6). With these data, a *k*_cat_ value of 0.88 ± 0.024 s^−1^ and a *k*_cat_/*K*_*m*_ value of 2.84 × 10^3^ M^−1^ s^−1^ were calculated.

10.1128/mBio.00092-17.3 FIG S2  MgCl_2_ dependency of MupP. The MurNAc 6P phosphatase activity of MupP in the absence (left) or presence (right) of MgCl_2_ was investigated by LC-MS in negative-ion mode. Shown are the EIC intensities (10^4^ counts/s [cps]) of MurNAc (*m/z* = 292.103; in orange) and MurNAc 6P (*m/z* = 372.070; in blue) with retention times of 5 and 17 min, respectively, on a ZIC hydrophilic interaction chromatography column. Download FIG S2, TIF file, 0.03 MB. Copyright © 2017 Borisova et al. 2017 Borisova et al. This content is distributed under the terms of the Creative Commons Attribution 4.0 International license.

10.1128/mBio.00092-17.4 FIG S3  Effects of temperature and pH on MupP activity. Experiments to determine MupP enzyme activity with MurNAc 6P as a substrate were performed as described in Materials and Methods. Graphs represent the effects of different temperatures (left) and pHs (right) on enzyme stability (black squares) and optimum activity (blue triangles). Relative MupP activity was determined by calculating the AUC of the EIC for MurNAc (*m/z* = 292.103) and is presented as a percentage. Experiments were done with three biological replicates, and values are presented as the mean ± the standard deviation in the GraphPad Prism 6 program. Download FIG S3, TIF file, 0.02 MB. Copyright © 2017 Borisova et al. 2017 Borisova et al. This content is distributed under the terms of the Creative Commons Attribution 4.0 International license.

10.1128/mBio.00092-17.5 FIG S4  MupP kinetic parameters. Samples for MupP kinetic experiments with MurNAc 6P substrate (0.0625 to 2 mM, left) or MurNAc standard curve determination (0.6 to 78 pmol, right) in 33 mM phosphate buffer (pH 7.6) with 10 mM MgCl_2_ in 20-µl total volumes were mixed with equal amounts of citrate-phosphate buffer, pH 3. Three-microliter portions of the 40-µl samples or standards were analyzed by HPLC-MS in negative-ion mode in accordance with reference 5. Data are presented as AUCs with an EIC baseline of 30 for MurNAc with *m/z* = 292.103. The MupP kinetic experiments were done in triplicate, and results are presented as mean values ± standard deviations. Download FIG S4, TIF file, 0.02 MB. Copyright © 2017 Borisova et al. 2017 Borisova et al. This content is distributed under the terms of the Creative Commons Attribution 4.0 International license.

**FIG 7:**
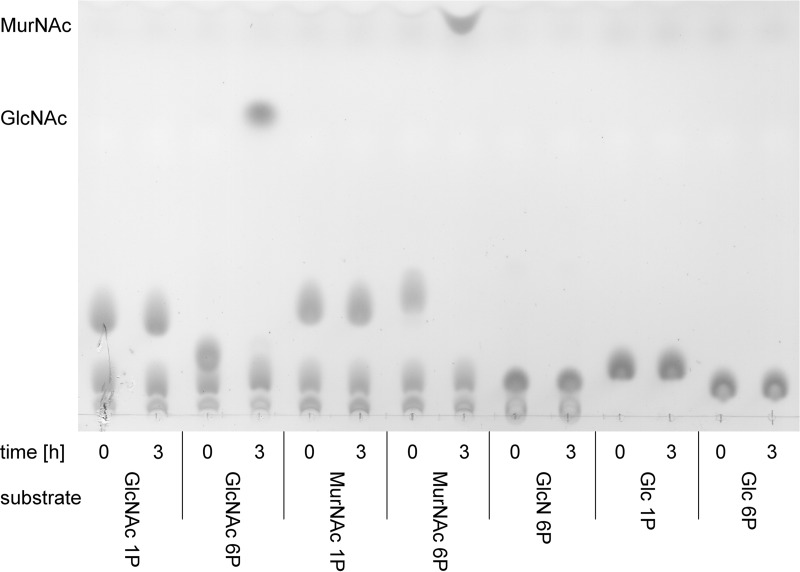
Substrate specificity of MupP. Twenty-five millimolar GlcNAc 1P, GlcNAc 6P, MurNAc 1P, MurNAc 6P, GlcN 6P, Glc 1P, or Glc 6P was incubated with 1.93 mM (50 mg/liter) recombinant MupP enzyme. Samples were loaded onto TLC plates at time zero and after 3 h of incubation at 37°C.

We also analyzed the substrate specificity of MupP. Therefore, a time course reaction was conducted with different sugar phosphates (25 mM) and very high enzyme concentrations (1.93 mM or 50 mg/liter) (Fig. 7). Neither α-1-phosphorylated MurNAc, GlcNAc, or glucose nor glucosamine 6P or glucose 6P was used as a MupP substrate. Of the phosphosugars tested, MupP showed detectable activity only with MurNAc 6P and GlcNAc 6P (Fig. 7). It should be noted, however, that GlcNAc 6P phosphatase activity was observed only with very large amounts of enzyme and a high substrate concentration. Using MS, we revealed that MupP is >100 times as fast with MurNAc 6P as with GlcNAc 6P (data not shown). Thus, we can note that MurNAc 6P is the preferred substrate of MupP.

## DISCUSSION

MupP (PP_1764) was identified in this study and characterized as a specific MurNAc 6P phosphatase in *P. putida* that is required for anabolic PG recycling and intrinsic fosfomycin resistance (Fig. 1). We used fosfomycin sensitivity testing in combination with multigenome homology analyses for the identification of *mupP* of *P. putida*. Among candidate genes that frequently co-occur in genomes with *amgK* and *murU* but were absent in *E. coli*, we found three members of the HAD superfamily (26) with reported phosphatase activity by using a chromogenic artificial substrate (27). Two of these phosphatases, PP_1764 and PP_1907, showed 29% (68 of 238 amino acids identical) and 30% (47/155), respectively, overall amino acid sequence identity with the 2-phosphoglycolate phosphatase Gph of *E. coli* when the basic local alignment search tool (28) was used. Gph is one of only a few HAD phosphatases that have been characterized so far (29). However, 2-phosphoglycolate is unlikely to be a substrate of PP_1764 and PP_1907, since a putative ortholog of *E. coli* Gph was identified in *P. putida* (PP_0416) that displayed 46% (114/250) overall amino acid sequence identity. Since fosfomycin hypersensitivity was expected for a *mupP* mutant, we constructed mutants with both genes deleted and tested their fosfomycin sensitivity. Δ*pp_1764* showed a pronounced fosfomycin-susceptible phenotype, similar to effects previously shown for *amgK* and *murU* mutants, suggesting interference with the anabolic recycling pathway (22, 23). Furthermore, we showed that Δ*pp_1764* cells accumulate large amounts of MurNAc 6P (>18-fold more than the wild type). Altogether, these results indicate that *pp_1764* encodes the missing MurNAc 6P phosphatase.

A very intriguing finding is that a *mupP* deletion also causes a 6-fold drop in UDP-MurNAc levels (as well as a 4-fold drop in UDP-GlcNAc, whereas the level of anhMurNAc, serving as an internal control, remained the same in the mutant and the wild type), which could explain the fosfomycin susceptibility of a Δ*mupP* mutant. Apparently, cells blocked within the anabolic recycling pathway have less UDP-MurNAc and are not able to compensate for the loss of UDP-MurNAc by upregulating *de novo* PGN biosynthesis. Thus, lower concentrations of fosfomycin are required to further reduce UDP-MurNAc levels by blocking MurA. UDP-GlcNAc levels also are severely reduced in the mutant, which likely is an indirect consequence of UDP-MurNAc depletion and the inability to fill up the UDP-GlcNAc pool. In this vein, it is very compelling that Thomas Bernhardt’s group independently discovered an ortholog of *mupP* (PA3172) in *P. aeruginosa* by genetic screening for mutants affected in AmpC β-lactamase induction. They showed that deletion of *mupP* or another gene of the PGN recycling pathway causes elevated expression of AmpC and hence increased resistance to β-lactam antibiotics, which was explained by reduced steady-state levels of UDP-MurNAc-pentapeptide (25).

We characterized MupP and showed that it is a remarkably specific phosphatase. MurNAc 6P is the preferred substrate of MupP, with a *K*_*m*_ of 310 μM. Among the other phosphosugars tested only GlcNAc 6P was cleaved; however, this required very large amounts of MupP enzyme (>1 µg). MupP is estimated to be 100-fold more active with MurNAc 6P than with GlcNAc 6P. MupP is rather slow (*k*_cat_ of 0.88 s^−1^), but it should be kept in mind that this kinetic parameter was obtained at 22°C because of low enzyme stability at higher temperatures. For other recycling enzymes (AnmK, AmgK, MurK, and MurQ), the kinetic parameters *K*_*m*_ and *k*_cat_ were reported to range from 180 to 1,200 μM and 5 to 6 s^−1^ at 25 to 37°C, respectively (19, 22, 30, 31).

The benefit of using two steps, dephosphorylation (MupP) and phosphorylation (AmgK), instead of a single phosphomutase reaction to convert MurNAc 6P to MurNAc α-1P is unclear. In virtually all bacteria, the phosphoglucosamine mutase GlmM catalyzes an equilibrium interconversion of glucosamine 6P and glucosamine α-1P (32). A possible explanation would be a detrimental effect of the accumulation of anhMurNAc on the cells, and thus, the conversion by MupP and AmgK could provide a benefit by rapidly shifting the reactions to the product side. Indeed, anhMurNAc may be harmful for *E. coli*, since it was shown that in an *anmK* mutant large amounts of the sugar were secreted into the medium (20). Further studies are required to investigate the possible toxic effect of anhMurNAc.

The AnmK-MurU recycling route, here named anabolic PG recycling, relies on the specific MurNAc 6P phosphatase MupP and is present in a great number of Gram-negative bacteria, including severe pathogens, e.g., *Neisseria*, *Bordetella*, *Burkholderia*, *Brucella*, *Pseudomonas*, and *Legionella* species, to name a few. This route renders these bacteria intrinsically resistant to fosfomycin, possibly because of a reduction of the UDP-MurNAc pool level, which also is consistent with the observed β-lactam resistance phenotype shown in the accompanying report (25). Thus, MupP and the entire anabolic recycling pathway may serves as a novel target for antibacterial agents, particularly in combination therapy against Gram-negative pathogens.

## MATERIALS AND METHODS

### Chemicals, sugars, enzymes, and oligonucleotides.

GlcNAc, glucosamine 6P (GlcN 6P), glucose 1P (Glc 1P), glucose 6P (Glc 6P), and ATP were purchased from Sigma-Aldrich (Darmstadt, Germany). MurNAc was from Bachem (Bubendorf, Switzerland), and GlcNAc 6P was from Carbosynth. GlcNAc 1P, MurNAc α-1P, and MurNAc 6P were generated by enzymatic synthesis (for a description of the production of these phosphosugars, see Text S1). Enzymes for DNA restriction and cloning were obtained from New England Biolabs (Ipswich, MA), and Gene JET plasmid miniprep and PCR purification kits, isopropyl-β-d-thiogalactopyranoside (IPTG), prestained protein molecular size markers (20 to 120 kDa), and fosfomycin discs (200 µg; Oxoid) were purchased from Thermo Fisher Scientific. Oligonucleotide primers were obtained from MWG Eurofins (Ebersberg, Germany) and are listed in Table S1.

10.1128/mBio.00092-17.1 TEXT S1  Method used to produce phosphosugars. Download TEXT S1, DOCX file, 0.1 MB. Copyright © 2017 Borisova et al. 2017 Borisova et al. This content is distributed under the terms of the Creative Commons Attribution 4.0 International license.

### Bacterial strains, plasmids, and growth conditions.

The bacterial strains and plasmids used in this study are summarized in Table S2. *E. coli* DH5α and BL21(DE3) cells were grown at 37°C and *P. putida* KT2440 cells were grown at 30°C in lysogeny broth (LB Lennox; 5 g/liter yeast extract, 10 g/liter tryptone, 5 g/liter NaCl) with continuous shaking at 160 rpm. When required, the LB medium was solidified with agar (1.5%, wt/vol). When appropriate, antibiotics were used at the following concentrations: kanamycin, 50 µg/ml for *E. coli* and 100 µg/ml for *P. putida*; gentamicin, 10 µg/ml for *E. coli* and *P. putida*.

### Enzymatic assays using TLC.

For the generation of *P. putida* cell extracts, an overnight culture was used to inoculate 2 liters of LB medium to an initial OD at 600 nm (OD_600_) of 0.1. Bacteria were grown to an OD_600_ of 1 and then harvested by centrifugation at 4°C. Bacterial pellets were resuspended in ice-cold Tris buffer (20 mM Tris-HCl, 300 mM NaCl, pH 7.6), cells were broken up by passing the suspension three times through an Emulsiflex-B15 (Avestin, Canada), and cell debris was removed by centrifugation for 45 min at 40,000 × *g* at 4°C. A protein concentration in the bacterial extract of 25 µg/µl was determined by the method of Bradford with bovine serum albumin as the standard. To test *P. putida* cell extracts for phosphatase activity, 10 µl of extract was added to 10 µl of 50 mM sugar phosphate substrates and the mixture was incubated at 37°C for 24 h. As a control, the extracts were incubated at 95°C for 5 min to heat inactivate proteins prior to addition to the phosphorylated sugar substrates. After incubation for 24 h, all samples were incubated at 95°C for 5 min and denatured protein was removed by centrifugation (2 min at 17,000 × *g*). Two 2.5-µl samples of cell extracts of wild-type *P. putida* were spotted onto a TLC plate (silica 60 F254; Merck, Darmstadt, Germany) and separated with the eluent butan-2-ol–CH_3_OH–NH_4_OH (25% in water)–water at 5:4:3:1 (vol/vol). The TLC plate was dried and subsequently processed with 5% sulfuric acid in methanol and charred at 180°C for about 5 min.

Similarly, MupP substrate specificity was analyzed by TLC. Different phosphosugars, MurNAc 6P, GlcNAc 6P, MurNAc 1P, GlcNAc 1P, GlcN 6P, Glc 1P, and Glc 6P, were used as the substrates for MupP. Fifty-microliter reaction mixtures, each with 25 mM substrate, 5 mM MgCl_2_ in Tris buffer (pH 7.6), and 2.5 µg (1.93 mM) of MupP enzyme were incubated at 37°C. Five-microliter samples were taken at 0 and 3 h, and two 2.5-µl portions were spotted onto a silica TLC plate as described above.

### Bioinformatic analysis.

The *Pseudomonas* Genome Database (Brinkman Lab, Simon Fraser University, Vancouver, BC, Canada) was used to compare protein sequences (33). The degree of conservation of the homologous proteins within their coding gene regions was examined by using the KEGG database and the gene clusters tool (34). To identify the MurNAc 6P phosphatase MupP, the distribution of putative homologous proteins was initially analyzed with the comparative microbial resource tool (http://cmr.jcvi.org/tigr-scripts/CMR/CmrHomePage.cgi) (35). In the meantime, the above-mentioned website is no longer supported by the Craig Venter Institute. Therefore, the bioinformatic analysis for *mupP* identification was conducted additionally with an alternative phylogenetic profiler for single genes provided by the Integrated Microbial Genomes & Microbiomes database (https://img.jgi.doe.gov/cgi-bin/w/main.cgi?section=PhylogenProfiler&page=phyloProfileForm). With that program, the annotated proteins in *Gammaproteobacteria* and *Betaproteobacteria* were checked for putative homologues of MupP. *P. putida* KT2440 was used as the reference genome, and a homology threshold of 30% amino acid sequence identity was set. Five different genomes were included in each analysis, and all of them code for both AmgK and AnmK. Additionally, the homologous proteins of *E. coli* MG1655 were excluded. The analysis was performed three times with different genomes. Putative phosphatases, namely, PP_1764, PP_1907, and PP_5147, were selected from the list of candidates as putative MupP candidates.

### Fosfomycin disc diffusion assay.

For fosfomycin susceptibility testing, solid agar LB plates (17.5 ml) were overlaid with 2.5 ml of soft agar containing 10 µl of overnight cultures of wild-type and mutant *Pseudomonas* strains. For a MupP complementation study, the *Pseudomonas* strains were transformed with the empty pUCP24 or p*mupP* plasmid and therefore the solid and soft agars were additionally supplemented with 10 µg/ml gentamicin. The plates were air dried for 10 min, and then 200-µg fosfomycin discs were added to each agar plate. After overnight incubation at 30°C, the inhibition zones were measured.

### Analysis of metabolites in cytosolic fractions of *P. putida* by MS.

Wild-type *P. putida* KT2440 cells and the respective Δ*mupP* mutant cells were grown to an OD_600_ of 1 in 200 ml of LB medium, and cytosolic extracts were generated as previously described (22). Dried samples were dissolved in 100 µl of water, and 3-µl amounts were subjected to LC-MS analysis with an UltiMate 3000 Rapid Separation LC system (Dionex) coupled to an electrospray ionization-time of flight mass spectrometer (MicrO-TOF II; Bruker) that was operated in negative-ion mode. Separation of metabolites was achieved with a Gemini C_18_ column (150 by 4.6 mm, 110 Å, 5 μm; Phenomenex) at 37°C with a flow rate of 0.2 ml/min in accordance with a previously described 45-min gradient program (22) that starts with 5 min of washing with 100% buffer A (0.1% formic acid, 0.05% ammonium formate in water), followed by a linear gradient over 30 min to 40% buffer B (acetonitrile). A 5-min hold at 40% B and a 5-min reequilibration step with 100% buffer A complete the process. The mass spectra of the samples investigated were created with Data Analysis (Bruker) and Prism 6 (GraphPad) software and are shown as TICs and EICs of metabolites presented as both measured and theoretical *m/z* values, if deviation of these values occurs. The theoretical *m/z* values of the metabolites investigated are 300.048 for GlcNAc 6P, 372.070 for MurNAc-6P, 292.103 for MurNAc, 274.093 for anhMurNAc, 678.095 for UDP-MurNAc, and 606.073 for UDP-GlcNAc.
