# Supplementary material for: The N-Acetylmuramic Acid 6-Phosphate Phosphatase MupP Completes the Pseudomonas Peptidoglycan Recycling Pathway Leading to Intrinsic Fosfomycin Resistance
Source: mBio. 2017 Mar 28;8(2):e00092-17. doi: 10.1128/mBio.00092-17 (PMC5371407; doi:10.1128/mBio.00092-17)
Supplement: TABLE S1 [file mbo002173259st1.docx]

**Table S1. List of oligonucleotides**

| **Primer** | **Sequence- 5'-3'*** | **Application** |  |
| --- | --- | --- | --- |
| pEXAp-for | **AGC CCA CTG CAA GCT** CGG CAT CAG AGC AGA TTG TA | amplification of pEX18Ap | |
| pEXAp-rev | **GAT AAA TCT GGA GC**C GGT GA | amplification of pEX18Ap | |
| pEX-km-for | **GCT CCA GAT TTA** **TC**A CAC GTC TTG AGC GAT TGT G | amplification of Km cassette from pKD13 | |
| pEX-km-rev | **AGC TTG CAG TGG GCT** TAC AT | amplification of Km cassette from pKD13 | |
| PP_1764-for | AGA CAT ATG CGT TTG CGA GCA GTA CTC TTC | generation of pJGK84 (pET29-*pp_1764*) | |
| PP_1764-rev | AGA GAA TTC AAA GCT TGC ACC CGC ACA GCG CGC TGT CGA T | generation of pJGK84 (pET29-*pp_1764*) | |
| PP_1764-EcoRI | AGT GAA TTC ACA TGA TCC AGA CCC TGC GC | generation of p*mupP* (pUCP24-*mupP*) | |
| PP_1764-BamHI | AGT GGA TCC GGT CAT CGG TCT GGG TTC A | generation of p*mupP* (pUCP24-*mupP*) | |
| up-pp_1764-for | GCA AGA AAG TGC TGG ACG TG | amplification of upstream region of *pp_1764* | |
| up-pp_1764-rev | **CAG ATC AGT TAT TCC TC**G CGC AGG GTC T | amplification of upstream region of *pp_1764* | |
| ds-pp_1764-for | **GAG GAA TAA CTG ATC TG**C CCG GAC CAC G | amplification of downstream region of *pp_1764* | |
| ds-pp_1764-rev | CGT AGA CCT CGT TCA GGT TGG | amplification of downstream region of *pp_1764* | |
| K-pp_1764-for | GCG AAG TTT CAG AGG ACT GTT CA | verification of JGK81 (∆*pp_1764*) | |
| K-pp_1764-rev | TAC AGG TAC ACC GGC ATG ATC TC | verification of JGK81 (∆*pp_1764*) | |
| up-pp_1907-for | GCG ACA CGT CTG GTC TGT TG | amplification of upstream region of *pp_1907* | |
| up-pp_1907-rev | **TCG TCT GCC ATG GAT CAG GGC G**CA CTC AGA | amplification of upstream region of *pp_1907* | |
| ds-pp_1907-for | **CGC CCT GAT CCA TGG CAG ACG A**AT GGA AA | amplification of downstream region of *pp_1907* | |
| ds-pp_1907-rev | TCT CCA TGG TGC CAA CAA AG | amplification of downstream region of *pp_1907* | |
| K-pp_1907-for | GGA GAA TTG CGA AGG TCA GG | verification of JGK71 (∆*pp_1907*) | |
| K-pp_1907-rev | CCT TGT GCT CAC CCG ATG TA | verification of JGK71 (∆*pp_1907*) | |

*restriction sites are underlined, overlapping regions of primer pairs are shown in bold. Km – kanamycin.
