# Supplementary material for: The N-Acetylmuramic Acid 6-Phosphate Phosphatase MupP Completes the Pseudomonas Peptidoglycan Recycling Pathway Leading to Intrinsic Fosfomycin Resistance
Source: mBio. 2017 Mar 28;8(2):e00092-17. doi: 10.1128/mBio.00092-17 (PMC5371407; doi:10.1128/mBio.00092-17)
Supplement: TABLE S2 [file mbo002173259st2.docx]

**Table S2. List of strains and plasmids**

| **Strains** | | **Genotype and cloning strategy*** | | **Reference or source** |
| --- | --- | --- | --- | --- |
| ***E. coli*** |  | | |  |
| DH5α | *supE44 hsdR17 recA1 endA1 gyrA96 thi-1 relA1* | | | New England Biolabs |
| BL21 (DE3) | F– *ompT* *hsd SB(rB–mB–) gal dcm* (DE3) | | | (6) |
| ***P. putida*** |  | | |  |
| KT2440 | parental strain, *mt-2 (r^-^ m^+^)* | | | (7) |
| JGK34 | ∆*anmK* (*pp_0434*) | | | (3) |
| JGK44 | ∆*amgK* (*pp_0405*) | | | (3) |
| JGK71 | Δ*pp_ 1907* | | | this study |
| JGK81 | ∆*mupP* (*pp_1764*) | | | this study |
| JGK82 | Δ*pp_1764* *-1907* (Δ*pp_1764* ∆*pp_1907*) | | | this study |
| **Plasmids** |  | | |  |
| pET-29b (+) | *E. coli* expression vector, Km^R^ | | | Novagen |
| pEX18Ap | gene replacement, suicide vector, *sacB*, Ap^R^ | | | (8) |
| pKD13 | template for amplification of Km^R^ casette, Km^R^, Ap^R^ | | | (9) |
| pEX18Km | gene replacement, suicide vector, *sacB*, Km^R^ | | | this study |
| pUCP24 | *E. coli – Pseudomonas* shuttle vector, Gm^R^ | | | (10) |
| pJGK81 | up/ds-*pp_1764* in pEXKm , SmaI, Km^R^ | | | this study |
| pJGK71 | up/ds-*pp_1907* in pEXKm, SmaI, Km^R^ | | | this study |
| pJGK84 | pET29*-mupP (pp_1764)* C-terminal His_6_, NdeI/HindIII, Km^R^ | | | this study |
| p*mupP* | pUCP24-*mupP* (*pp_1764*) in EcoRI/BamHI, Gm^R^ | | | this study |
| *Km - kanamycin; Gm - gentamycin; Ap - apramycin; | | |  | |
