# Supplementary figures and images for: The N-Acetylmuramic Acid 6-Phosphate Phosphatase MupP Completes the Pseudomonas Peptidoglycan Recycling Pathway Leading to Intrinsic Fosfomycin Resistance
Source: mBio. 2017 Mar 28;8(2):e00092-17. doi: 10.1128/mBio.00092-17 (PMC5371407; doi:10.1128/mBio.00092-17)

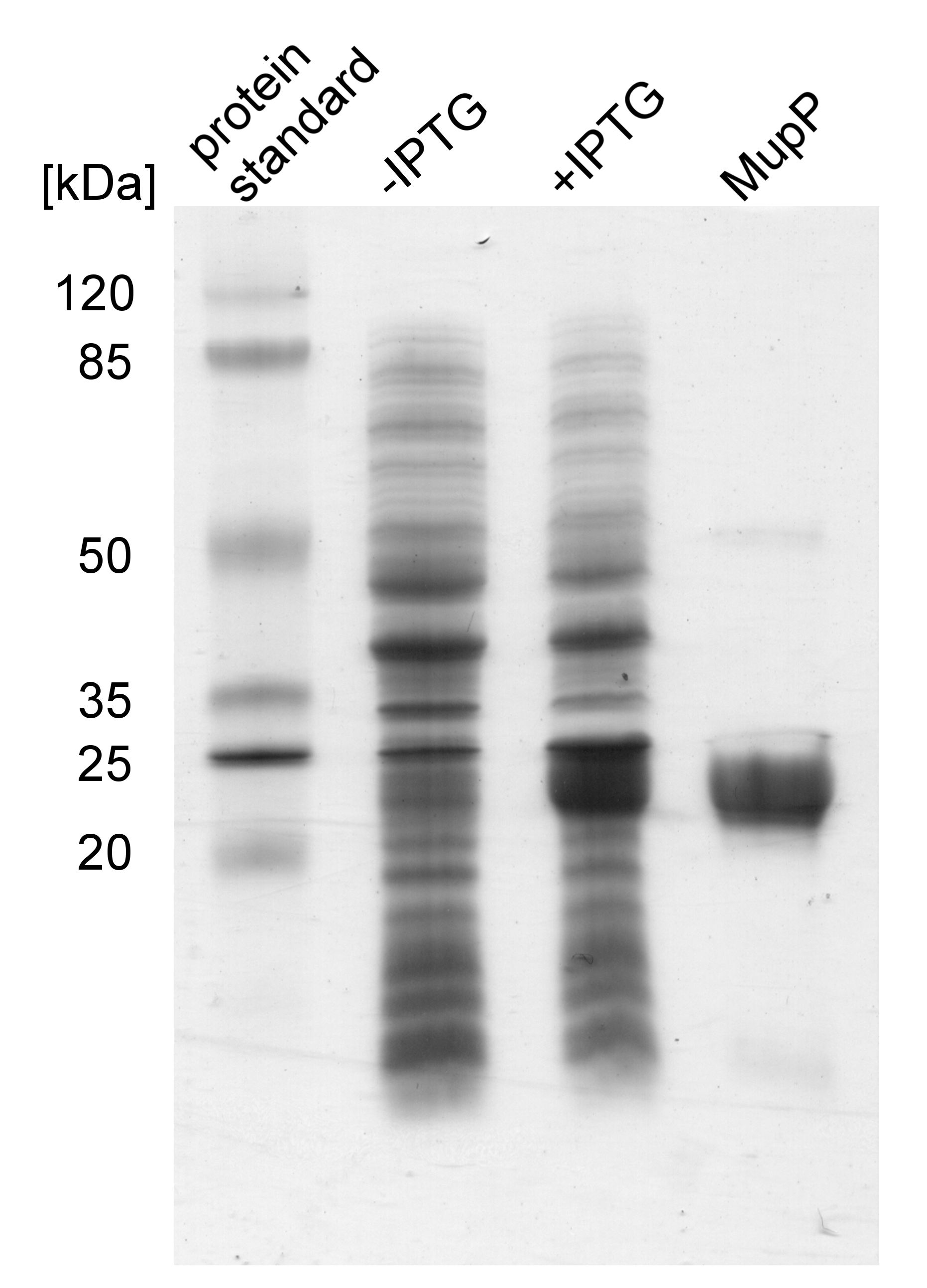

Supplement: FIG S1 [file mbo002173259sf1.tif]
