# Supplementary material for: The N-Acetylmuramic Acid 6-Phosphate Phosphatase MupP Completes the Pseudomonas Peptidoglycan Recycling Pathway Leading to Intrinsic Fosfomycin Resistance
Source: mBio. 2017 Mar 28;8(2):e00092-17. doi: 10.1128/mBio.00092-17 (PMC5371407; doi:10.1128/mBio.00092-17)
Supplement: TEXT S1 [file mbo002173259s1.docx]

**Supplemental Material for:**

**The *N*-acetylmuramic acid 6-phosphate phosphatase MupP completes the Pseudomonas peptidoglycan recycling pathway leading to intrinsic fosfomycin resistance**

Marina Borisova, Jonathan Gisin, and Christoph Mayer#

Department of Biology, Interfaculty Institute of Microbiology and Infection Medicine, University of Tübingen, Tübingen, Germany

**Running title:** MurNAc 6-phosphate phosphatase MupP

#Address correspondence to Christoph Mayer, christoph.mayer@uni-tuebingen.de

M.B. and J.G. contributed equally to this work

**Construction of deletion mutants.** Primers, strains, and plasmids used in this study are listed in **Tables S1 and S2 in the supplemental material**. Suicide vector pEX18Km, which carries a kanamycin resistance cassette (Km^R^) and the *sacB* gene conferring sucrose sensitivity, was generated using the PIPE method (1). Therefore, two PCR products with overlapping ends were generated, the vector backbone of pEX18Ap was amplified using primer pair pEXAp-for and pEXAp-rev, and the kanamycin resistant gene of plasmid pKD13 was amplified using primer pair pEX-km-for and pEX-km-rev. The PCR fragments were mixed (5 µl each) and the mixture was transformed in DH5α cells. The plasmid pEX18Km was extracted from kanamycin-resistant transformants.

For the deletion of *pp_1764* of *P. putida*, regions on the chromosome upstream (primer pair up-pp_1764-for and up-pp_1764-rev) and downstream (primer pair ds-pp_1764-for and ds-pp_1764-rev) were amplified by PCR. The PCR products, containing complementary overhanging ends, were extracted from an agarose gel using a gel extraction kit (Thermo Fisher Scientific) and joined together by assembly PCR using primers up-pp_1764-for and ds-pp_1764-rev. The product finally was cloned into the SmaI restriction site of the vector pEX18Km yielding recombinant plasmid pEX18Km-∆*pp_1764*, named pJGK81. Kanamycin-resistant *E. coli* cells, carrying plasmid pJGK81, which have the up- and downstream region inserted into the *lacZα*, were identified by blue-white screening on X-gal (5-Bromo-4-chloro-3-indolyl β-D-galacto-pyranoside) and the DNA sequence was confirmed by sequencing (MWG Eurofins). Isolated pJGK81 plasmid was transformed in *P. putida* KT2440 wild type cells by electroporation as previously described (2) and transconjugants that have undergone a single recombination event were selected on LB plates supplemented with 100 µg/ml kanamycin. Kanamycin-resistant *P. putida* clones were further subjected to counter selection on LB plates containing 5% sucrose. Single colonies were tested for kanamycin-sensitivity and the *pp_1764* gene deletion was identified by colony PCR using primers K-pp1764-for and K-pp1764-rev (data not shown). The resulting markerless mutant strain was named JGK81.

Mutant *∆pp_1907* and double mutant Δ*pp_1764-1907* (Δ*mupP* ∆*pp_1907)* were obtained by a similar procedure. For *∆pp_1907* the primer pair up-pp_1907-for and up-pp_1907-rev was used for amplification of upstream regions and primers ds-pp_1907-for and ds-pp_1907-rev for amplification of downstream regions. Recombinant plasmid pEX18Km-∆*pp_1907*, named pJGK71, was transformed into *P. putida* KT2440 and JGK81 (∆*mupP*)*.* Deletion of gene *pp_1907* in the Δ*pp_1907* (JGK71) and Δ*pp_1764* Δ*pp_1907* (JGK82) mutants was verified by primers K-pp_1907-Fw and K-pp_1907-Rev (data not shown).

**Construction of plasmids for complementation and expression of MupP.** For complementations, the gene *pp_1764* (*mupP*) of *P*. *putida* KT2440 was cloned into the pUCP24 plasmid and constitutively expressed under the control of the *lac* promoter. Therefore, genomic DNA from *P*. *putida* KT2440 was isolated and the *pp_1764* gene, including its native ribosome binding site was amplified using primer pair PP_1764-EcoRI and PP_1764-BamHI. The obtained 737 bp PCR product was digested with BamHI and EcoRI restriction enzymes and cloned in the multiple cloning site of the pUCP24 plasmid. Obtained pUCP24-*mupP* plasmid, named p*mupP* was isolated from gentamycin–resistant DH5α cells and DNA sequence was verified by enzyme restriction and sequencing (MWG Eurofins). The plasmid was further used to electroporate *P. putida* wild type and *∆pp_1764* cells and successful transformants were selected on LB plates, supplemented with 10 µg/ml gentamycin.

For heterologous overexpression in E. coli, *pp_1764* (mupP) gene was cloned in pET-29b (+) plasmid as a recombinant protein with a C-terminal His_6_ tag. *P. putida* KT2440 genomic DNA was used to amplify a DNA fragment by PCR using PP_1764-for and PP_1764-rev primers. The PCR fragment was ligated into the pET-29b (+) expression vector by T4 DNA ligase using NdeI and HindIII restriction sites and transformed in chemically competent DH5α cells. The pET29-*mupP* (pJGK84) recombinant plasmid was isolated from kanamycin-resistant *E. coli* cells and DNA sequence was verified by sequencing. The pET29-*mupP* plasmid was further transformed into BL21 (DE3) cells for MupP heterologous overexpression and was expressed under the control of the IPTG (isopropyl-β-d-thiogalactopyranoside)-inducible T7 promoter.

**Enzymatic production of phosphorylated GlcNAc and MurNAc sugars.** MurNAc α-1P and GlcNAc α-1P sugars were generated using the anomeric kinase AmgK from *P. putida* (3), which phosphorylates MurNAc and GlcNAc at the anomeric carbon. MurNAc 6P and GlcNAc 6P were generated by phosphorylation of MurNAc and GlcNAc by the kinase MurK from *Clostridium acetobutilicum,* which phosphorylates the hydroxyl group at C6, according to described protocols (4, 5). Briefly, a mixture of 50 mM GlcNAc or MurNAc, 100 mM ATP, 10 mM MgCl_2_ in 100 mM Tris-HCl buffer (pH 7.6), was incubated overnight at 37°C with 25 µg of the AmgK or MurK kinases in a total reaction volume of 500 μl. To remove the enzyme in the reaction, the samples were filtered through centrifugal filter devices with a 10 kDa cut-off filter (Amicon Ultra, Millipore). The enzyme-free filtrate, containing the phosphorylated sugars, ATP, ADP and MgCl_2_ was used further for testing substrate specificity of the putative sugar phosphatase in *P. putida* cells by thin layer chromatography (TLC). For the enzyme kinetics experiments by LC-MS the phosphosugars were further purified using HPLC according to published protocols (4, 5).

**Heterologous expression and purification of MupP-His_6_.** MupP enzyme from *P. putida* was overproduced in E. coli BL21 (DE3) carrying pET29-*mupP* (pJGK84) plasmid. Bacteria were grown under vigorous shaking at 37°C in 2 liters of LB medium supplemented with 50 μg/ml kanamycin, starting from a 1% inoculum of an overnight culture. The expression of MupP was induced by the addition of IPTG at 0.1 mM final concentration after the culture reached an OD_600nm_ of about 0.7. Three hours after IPTG induction the cells were harvested by centrifugation at 4°C. The cell pellet was resuspended in 30 ml buffer A (20 mM Tris-HCl, 300 mM NaCl, 1 mM DTT, pH 7.6) and broken up by passing the suspension three times through an Emusiflex-B15 (Avestin, Canada). The soluble proteins were separated from the cell debris by centrifugation with 40,000 g for 1 h at 4°C. The His-tagged protein was purified from the soluble cell extract by Ni^2+^ affinity chromatograpy system Äkta Purifier on a 1 ml His-Trap column (GE Healthcare). A linear gradient over 30 min from 96% buffer A with 4% buffer B (20 mM Tris-HCl, 300 mM NaCl, 500 mM imidazole,1 mM DTT pH 7.6) to 100% buffer B was used to elute the MupP enzyme from the His-Trap column. The elution fractions with UV_280nm_ active substances were analyzed on 12% SDS-PAGE gel and the MupP containing fractions (exact mass of 25,892 Da) were collected and subjected to an additional chromatography with HP 26/10 desalting column and 100% buffer A to remove the imidazol. Protein purity was controlled by SDS-PAGE. An extinction coefficient at 280 nm of 15470 M^-1^ cm^-1^ was calculated with the ExPASy ProtParam tool to determine the concentration of MupP. Protein in 20% glycerol was stored at -80°C.

**pH and temperature stability and optima of MupP.** If not otherwise stated, experiments for the determination of the MupP stability and optima were conducted at 22°C in 50 µl reactions containing 33 mM phosphate buffer pH 7, with 10 mM MgCl_2_, 1 mM MurNAc 6P, and 8.7 ng (6.72 nM) of the recombinant MupP enzyme.

For determination of temperature stability, the MupP enzyme was pre-incubated for 30 min at different temperatures (4, 22, 37, 45, 55 and 65°C) in a total volume of 47.2 µl. Afterwards, the temperature in all samples was brought to 22°C and kept at this temperature for 10 minutes. The enzyme reactions were started by adding 2.8 µl of MurNAc 6P substrate (18 mM). After 5 minutes, the reaction was stopped by addition of 50 µl of 200 mM of citrate-phosphate (McIlvaine) buffer pH 3. For determination of the temperature optimum, MurNAc 6P (1 mM concentration in 50 µl final volume) was pre-incubated in phosphate buffer with MgCl_2_ at different temperatures (see above) for 10 min and then the reaction was started by adding MupP to each sample. After 5 min incubation at the respective temperatures the enzyme reactions were stopped by adding equal volumes of 200 mM Mclvaine buffer pH 3.

For determination of pH stability, 10 µg of the MupP enzyme was incubated for 30 min at 22°C in 200 µl of the following buffers (50 mM): pH 2 (Clark and Lubs), pH 3 to 6 (acetate), pH 5 to 8 (phosphate), pH 9 to 10.7 (carbonate). Afterwards, the enzyme samples were diluted in 33 mM phosphate buffer pH 7, with MgCl_2_, and a volume containing 8.7 ng of MupP was removed from each sample and added to a new reaction tube containing MurNAc 6P (final concentration of 1 mM). After 5 min, the enzyme reactions were stopped with citrate-phosphate buffer. For determination of the pH optimum, MurNAc 6P (1 mM final concentration) was solved in different buffer (50 mM, 50 µl final volume) with pH 2 to 10.7 supplemented with 10 mM MgCl_2_. After addition of the MupP enzyme and incubation for 5 min, 50 µl of McIlvaine buffer pH 3 was added to each sample. 3-µl of each 100 µl sample was analyzed by liquid chromatography-mass spectrometry (LC-MS), to determine the MurNAc released by MupP.

**Determination of MupP enzyme kinetics by mass spectrometry.** MupP kinetic experiments were conducted in 20 µl reactions containing 33 mM phosphate buffer pH 7.6, with 10 mM MgCl_2_, 0.0625 mM to 2 mM MurNAc 6P, and 10 ng (19.31 nM) of the MupP. After 2.5 min incubation at 22°C, 20 µl of McIlvaine buffer (200mM, pH 3) was added to stop the enzyme reaction. 3 µl of each sample was analyzed by LC-MS, to determine the MurNAc released by MupP at different concentration of MurNAc 6P.

LC-MS analysis was performed as described (3). The mass spectra of the investigated samples are presented as extracted ion chromatograms (EIC) for MurNAc (m/z theoretical = 292.103) created using Data Analysis (Bruker) and Prism 6 (GraphPad) software. The area under the curve for the EIC for MurNAc with retention time about 5 min was obtained by using Prism 6 and a baseline set to a value of 30.

**Supplemental figure legends**

**FIG S1. Analysis of MupP overexpression and purity by SDS-PAGE and Coomassie brilliant blue staining.** The protein was overproduced in *E. coli* BL21 (DE3) cells carrying pET29b-*mupP* (p*mupP*) plasmid. Lane 1, protein 20-120 kDa standard; lane 2, *E. coli* cell extract before IPTG induction; lane 3, *E. coli* cell extract after 0.1 mM IPTG induction for MupP overexpression; lane 4, 25 μg of purified MupP enzyme. The exact size of MupP is 25,892 kDa.

**FIG S2. MgCl_2_ dependency of MupP.** MurNAc 6P phosphatase activity of MupP was investigated in the absence (left) or presence (right) of MgCl_2_ by liquid chromatography-mass spectrometry in negative ion mode. Shown are extracted ion chromatograms (EIC) intensity x 10^4^ counts per second (cps) for MurNAc (m/z = 292.103) in orange and MurNAc 6P (m/z = 372.070) in blue with retention times on the ZIC-HILIC column of 5 min and 17 min, respectively.

**FIG S3. Effect of temperature and pH on MupP activity.** Experiments to determine MupP enzyme activity using MurNAc 6P as a substrate were performed as described in material and methods. Graphs represent the effect of different temperature (left) and pH (right) on enzyme stability (black squares) and optimum (blue triangles). Relative MupP activity is presented by calculating the area under the curve of the extracted ion chromatograms (EIC) for MurNAc (m/z = 292.103) and presenting in %. Experiments were done in three biological replicates and values are presented as mean ± standard deviation in GraphPad Prism 6 program.

**FIG S4. MupP kinetic parameters.** Samples for MupP kinetic experiments using MurNAc 6P substrate (0.0625 mM to 2 mM, left) or MurNAc standard curve (0.6 to 78 pmol, right) in 33 mM phosphate buffer pH 7.6, 10 mM MgCl_2_ in 20 µl total volumes were mixed with equal amounts of citrate-phosphate buffer, pH 3. 3-µl from the 40 µl samples or standards was analyzed by HPLC-MS in negative ion mode, according to (5). Data are presented as area under the curve (AUC) with a baseline of 30 of extracted ion chromatogram (EIC) for MurNAc with m/z = 292.103. The MupP kinetic experiments were done in triplicates and values are presented as mean ± standard deviation.

**Supplemental tables**

**Table S1. List of oligonucleotides**

**Table S2. List of strains and plasmids**

**References**

1. **Klock HE, Lesley SA.** 2009. The Polymerase Incomplete Primer Extension (PIPE) method applied to high-throughput cloning and site-directed mutagenesis. Methods Mol Biol **498:**91-103.

2. **Choi KH, Kumar A, Schweizer HP.** 2006. A 10-min method for preparation of highly electrocompetent Pseudomonas aeruginosa cells: application for DNA fragment transfer between chromosomes and plasmid transformation. J Microbiol Methods **64:**391-397.

3. **Gisin J, Schneider A, Nägele B, Borisova M, Mayer C.** 2013. A cell wall recycling shortcut that bypasses peptidoglycan de novo biosynthesis. Nat Chem Biol **9:**491-493.

4. **Reith J, Berking A, Mayer C.** 2011. Characterization of an *N*-acetylmuramic acid/*N*-acetylglucosamine kinase of *Clostridium acetobutylicum*. J Bacteriol **193:**5386-5392.

5. **Unsleber S, Borisova M, Mayer C.** 2017. unpublished.

6. **Studier FW, Moffatt BA.** 1986. Use of bacteriophage T7 RNA polymerase to direct selective high-level expression of cloned genes. J Mol Biol **189:**113-130.

7. **Bagdasarian M, Lurz R, Ruckert B, Franklin FC, Bagdasarian MM, Frey J, Timmis KN.** 1981. Specific-purpose plasmid cloning vectors. II. Broad host range, high copy number, RSF1010-derived vectors, and a host-vector system for gene cloning in *Pseudomonas*. Gene **16:**237-247.

8. **Hoang TT, Karkhoff-Schweizer RR, Kutchma AJ, Schweizer HP.** 1998. A broad-host-range Flp-FRT recombination system for site-specific excision of chromosomally-located DNA sequences: application for isolation of unmarked Pseudomonas aeruginosa mutants. Gene **212:**77-86.

9. **Datsenko KA, Wanner BL.** 2000. One-step inactivation of chromosomal genes in *Escherichia coli* K-12 using PCR products. Proc Natl Acad Sci USA **97:**6640-6645.

10. **West SE, Schweizer HP, Dall C, Sample AK, Runyen-Janecky LJ.** 1994. Construction of improved *Escherichia-Pseudomonas* shuttle vectors derived from pUC18/19 and sequence of the region required for their replication in *Pseudomonas aeruginosa*. Gene **148:**81-86.
